# Evaluation of the impact of reducing national emissions of SO_2_ and metals in Poland on background pollution using a bioindication method

**DOI:** 10.1371/journal.pone.0192711

**Published:** 2018-02-23

**Authors:** Wojciech Dmuchowski, Dariusz Gozdowski, Aneta H. Baczewska-Dąbrowska, Piotr Dąbrowski, Barbara Gworek, Irena Suwara

**Affiliations:** 1 Warsaw University of Life Sciences–SGGW, Nowoursynowska, Warsaw, Poland; 2 Polish Academy of Sciences Botanical Garden–Center for Conservation of Biological Diversity, Warsaw, Poland; Natural Environment Research Council, UNITED KINGDOM

## Abstract

Changes in environmental pollution by S, Cd, Cu, Pb and Zn in 2006–2014 were evaluated using a bioindication method. This method was based on measurements of pollutants in Scots pine (*Pinus sylvestris* L.) needles. The measurements were performed in the Chojnowskie Forests, a region recognized as a background area for central Poland. The changes in the contents of sulfur (S) and metals in needles were not comparable with the changes in the global emissions of the pollutants in Poland. On average, the pollution level in the study area decreased by 9.9% for S, 61.4% for Pb, 22.5% for Cd, 11.7% for Zn and 10.4% for Cu. During the same period, global emissions in Poland decreased by 38.1% for S, 8.0% for Pb, 63.2% for Cd, 11.7% for Zn and 14.0% for Cu. Therefore, the differences in the changes in emissions and the needle contents of each element should be examined separately which was not a goal of this study. However, the discrepancy between these results did not prevent the use of bioindication methods. Evaluation of pollutant contents in plants reflected their incorporation in biological processes rather than air or soil pollution levels.

## Introduction

Bioindication methods offer an opportunity for significant intensification of studies on air pollution, especially on the content of trace elements. Biomonitoring in a general sense can be defined as the use of plants and animals with the objective of gaining quantitative and qualitative information on certain characteristics of the biosphere [1–4].

Measurement of contaminant accumulation in Scots pine needles has been used as a biomonitoring method for entire countries, industrial regions, administrative units and single emission sources (Table 1) [5–86]. Measurement of sulfur and heavy metal concentration levels in Scots pine needles gained a recommendation from the United Nations Environment Program as a standard method for the assessment of environmental contamination [87].

**Table 1:** A summary of some relevant literature concerning of the use Scots pine needles in the biomonitoring of environmental pollution.

Area	Country	Elements	Authors
industrial	Great Britain	S	Farrar et al, 1977 [5]
still mill	Poland	S	Grodzińska, 1977 [6]
industrial	Scotland	S	Malcolm and Garforth, 1977 [7]
industrial	Finland	Fe, Ti, V, Zn	Laaksovirta and Olkkonen, 1979 [8]
background	Slovakia	F, As, Cd, Pb	Maňkowska, 1980 [9]
industrial	Finland	S	Lehtiö et al., 1980 [10]
urban	Finland	S	Soikkeli, 1981 [11]
Zn smelter	Poland	Zn, Pb, Cd, Fe	Niemtur, 1981 [12]
background	Finland, Poland	10 elements	Molski and Dmuchowski, 1990 [13]
urban	Finland	Al, Cd, Fe, Zn	Vilkka et al., 1990 [14]
industrial	Finland	S	Manninen et al., 1991 [15]
industrial	Germany	S	Polle et al., 1994 [16]
forest	Finland, Russia	S	Lumme et al., 1995 [17]
forest	Great Britain	14 elements	Innes, 1995 [18]
industrial	Finland, Russia Lapland	S	Raitio et al., 1995 [19]
background	Finland	11 elements	Palomäki and Raitio, 1995 [20]
whole country	Poland	S, As, Cd, Cu, Pb, Zn	Dmuchowski and Bytnerowicz, 1995 [21]
Cu-Ni smelter	Finland	Cu	Helmisaari et al., 1995 [22]
industrial	Finland, Russia Lapland	S, Cu, Ni	Poikolainen, 1997 [23]
industrial	Finland, Russia Lapland	S	Manninen et al., 1997 [24]
steel mill	Poland	16 elements	Kurczyńska et al., 1997 [25]
fertilizer factory	Poland	18 elements	Giertych et al., 1997 [26]
mountains	Poland	17 elements	Migaszewski et al., 1998 [27]
Cu-Ni smelter	Finland Lapland	S, Cu, Fe, Ni, Zn	Rautio et al., 1998 [28]
fertilizer factory	Poland	15 elements	Oleksyn et al., 1999 [29]
Cu-Ni smelter	Russia Lapland	34 elements	Steinnes et al., 2000 [30]
fertilizer factory	Poland	F	Karolewski et al., 2000 [31]
oil refinery	Finland	S	Manninen and Huttunen, 2000 [32]
Cu-Ni smelter	Russia, Lapland	11 elements	Koptsik et al., 2001 [33]
industrial	northern Europe	38 elements	Reimann et al., 2001 [34]
industrial	northern Finland	S	Pöykiö and Torvela, 2001 [35]
urban	Nigeria	S	Ayodele and Ahmed, 2001 [36]
mountains	Poland	PAHs, S, Cd, Hg, Pb, Zn	Migaszewski et al., 2002 [37]
rural	Spain	23 elements	Penuelas and Filella, 2002 [38]
industrial	Finland, Russia Lapland	S, Cu, Fe, Ni	Lamppu and Huttunen, 2003 [39]
forest	Germany	S	Schulz and Härtling, 2003 [40]
urban	Turkey	Cu, Pb, Zn	Yilmaz and Zengin, 2004 [41]
Cu–Ni smelter	Finland	Cu, Fe, Ni, Zn	Nieminen et al., 2004 [42]
fertilizer factory	Poland	15 elements	Karolewski et al., 2005 [43]
background	Finland	S, Al	Luyssaert et al., 2005 [44]
U-mining heap	Germany	U	Thiry et al., 2005 [45]
industrial	Russia, Siberia	S	Afanasyeva et al., 2005 [46]
industrial	Poland	8 metals	Samecka-Cymerman et al., 2006 [47]
industrial	Poland	Hg	Szynkowska and Pawlaczyk, 2007 [48]
industrial	Finland	14 elements	Fältmarsch et al., 2007 [49]
industrial	Russia, Siberia	Cd, Cu, Hg., Pb, Zn	Afanasieva et al., 2007 [50]
background	Poland	S, 6 metals	Gałuszka, 2007 [51]
background	Slovakia	S	Maňkovská and Oszlányi, 2008 [52]
Cu–Ni smelter	Russia, Lapland	S, Cd, Cu, Ni, Pb, Zn	Shcherbenko et al., 2008 [53]
industrial	Poland	F, S	Sochacka et al., 2009 [54]
background	northern Sweden	S, Fe, Mn, Zn	Ladanai et al., 2010 [55]
mountains	Turkey	S	Yucel and Guner, 2010 [56]
industrial	Finland	S	Pöykiö et al., 2010 [57]
background	Russia, Siberia	S, F, 7 metals	Mikhailova et al., 2011 [58]
Zn-Pb smelter	Poland	Cd, Pb	Dmuchowski et al., 2011 [59]
Zn-Pb smelter	Poland	As	Dmuchowski et al., 2011 [60]
Cu-Ni smelter	Russia, Lapland	S, Co, Cu, Ni, Fe	Pridacha et al., 2011 [61]
background	Poland	Cu, Fe, Mn, Zn	Parzych and Jonczak, 2013 [62]
urban	Norway	8 metals	Przybysz et al., 2014 [63]
Zn-Pb smelter	Poland	Zn	Dmuchowski et al., 2013 [64]
Zn smelter	Poland	Cd, Cu, Pb, Zn	Chudzinska et al., 2014 [65]
industrial	Poland	Cd, Cu, Pb, Zn	Chudzinska et al., 2014 [66]
Cu-Ni smelter	Russia, Lapland	S, 6 metals	Sukhareva and Lukina, 2014 [67]
Ni smelter	Finland, Russia, Norway,	17 elements	Rautio and Poikolainen, 2014 [68]
urban	Poland	Cu, Ni	Parzych and Jonczak, 2014 [69]
hard coal, lignite, S mine	Poland	Cd, Cu, Pb, Zn	Pietrzykowski et al., 2014 [70]
highway	Norway	25 elements	Mori et a., 2015 [71]
background	Norway	7 metals	Gjengedal et al., 2015 [72]
urban	Poland	Cd, Pb, Zn	Mazur et al., 2015 [73]
industrial	Poland	Cd, Pb, Zn	Pająk et al., 2015 [74]
industrial	southern Europe	6 metals	Andráš et al., 2016 [75]
urban	Poland	9 metals	Kosiorek et al., 2016 [76]
industrial	Poland	Cd, Fe, Mn, Pb, Zn	Kandziora-Ciupa et al., 2016 [77]
background	Czech, Sweden, Slovakia	16 elements	Holt et al., 2016 [78]
urban	Poland	Cd, Cr, Pb, Zn	Baczewska et al., 2016 [79]
background	Russia (Yakutia)	16 elements	Popova, 2016 [80]
whole country	Germany	As, Cr, Cu, Ni, Zn	Nickel and Schröder, 2016 [81]
Pb-Zn smelter	Poland	Pb, Zn, Cd, Cu, Cr	Pająk et al., 2017 [82]
chlor-alkali	Czech	Hg	Navrátil et al., 2017 [83]
urban	Poland	6 metals	Parzych et al., 2017 [84]
Al smelter	Russia (Siberia)	PAHs, F, S, 9 metals	Kalugina et al., 2017 [85]
chlor-alkali	Poland	14 metals	Klink et al., 2017 [86]

In Poland, measurements of air pollution via the measurement-and-control approach are mainly conducted in cities within the framework of the State Environmental Monitoring. In agro-forest areas, the number of measuring points is negligible. Application of bioindication methods provides the possibility of a significant intensification of studies into environmental pollution.

Sulfur dioxide air pollution is considered one of the main causes of forest decline in the northern hemisphere [5,88–91]. Several studies have shown that the sulfur content in Scots pine needles is correlated with the concentration of SO_2_ in the air [18–19,24,92].

The needles of Scots pine have been used to detect the deposition, accumulation and distribution of heavy metal pollution. Although it is often difficult to determine the sources of the heavy metals [93], trees can be used as effective biomonitors for detecting low concentrations of pollutants of both soil and atmospheric origin.

Trees are not the best indicators for monitoring air pollution when compared to lower plants (lichens or mosses) [94,95]. However, their one great advantage is that they are long-lived, so that a repetition of the investigation is possible after a few decades, as was conducted by Ballach and Wittig [96]. Thus, trees can be sampled systematically with standardized sampling and analytical techniques for comparative monitoring of the time-trend distribution of trace elements [97]. In addition, trees are usually easier to identify than lower plants [98]. They provide useful data for the design of international networks for deposition monitoring, and they greatly facilitate the analytical determination of trace elements.

Sulfur is emitted into the atmosphere mainly as sulfur dioxide (SO_2_). Emissions of dimethyl sulfide (H_2_S) are of marginal significance. The major anthropogenic source of sulfur dioxide emissions is the sulfur content of fossil fuels released by combustion, mainly hard coal and lignite. In addition, some sulfur is produced in petroleum refining, the smelting of sulfidic ores in the production of heavy metals, and the production of sulfuric acid, paper and sulfur. Natural fluxes of sulfur originate from volcanoes and from the biological and photochemical production in the oceans of volatile sulfur gases, notably dimethyl sulfide. Comparatively small amounts of sulfur are also emitted from forest fires, soils and vegetation, sulfur springs and sea salt. Emissions from natural sources are negligible outside those areas near active volcanoes [99–100].

Global sulfur dioxide emissions increased from 2,447 Gg in 1850 to 115,507 Gg in 2005. This emissions growth was observed in every region of the world up to the 1970s–1980s. Subsequently, there has been a substantial decrease in emissions in western Europe, the USA, Canada and Japan. In eastern Europe, a decrease in emissions has been recorded, starting in 1990–2000; emissions decreased in China and Russia, starting in 2005. Only in India, which has a significant share of global emissions, are emission levels still rising. The fall in global emissions started in 2005 [100–102].

In 2014 in the European Union (EU), the production of energy constituted 91% of overall SO_2_ emissions [103]. These overall emissions dropped by 81.6%: from 16.800 Gg in 1995 to 3.083 Gg in 2014 (Table 2). In Poland, the emissions fell by 65.2%: from 2.300 Gg in 1995 to 800 Gg in 2014. The emissions of SO_2_ in Poland constituted 11.9% of the overall EU emissions in 1990, and 26.0% in 2014, meaning that Poland was the highest emitter of SO_2_ in the EU. The second heaviest polluter, Germany, emits less than half as much [103]. The reason for such a high share in emissions for Poland (Table 3) is the use of coal as the main energy source. Coal contributes to more than 82% of overall energy production, while the share of renewable energy amounts to only 12%. In the years 1995–2014, the consumption of coal decreased by only 19.0% [104–105`].

**Table 2:** Decrease in SO_2_ and metals emissions in the European Union (EU-28) and Poland in the years 1995–2014 [103].

pollutant	EU-28				Poland			
	1995	2006	2014	Change (%) 1995–2014	1995	2006	2014	Change (%) 1995–2014
SO_2_ (Gg)	16 800	7 529	3 083	81.6	2 300	1 292	800	65.2
Cd (Mg)	152	113	62	59.2	27	38	14	48.1
Cu (Mg)	3 390	3 718	3 582	-5.7	361	379	326	9.7
Pb (Mg)	11 001	2 930	1 925	82.5	605	562	517	14.5
Zn (Mg)	8 915	7 384	6 822	23.5	1 779	1 547	1 366	23.2

**Table 3:** Coal consumption for energy purposes in Poland in the years 1995–2014 [104,105].

	1995	2006	2014	Change (%) 2006–2014
Hard coal	108 301	83 693	73 125	-12.6
Lignite	63 355	60 801	65 933	8.4
Total	171 656	144 494	139 058	-3.8

Contamination of the environment with heavy metals is primarily related to human activities. The sources of emissions are coal fuels (power stations, boiler houses, households), industry (especially metallurgy), transport and the use of sewage drift, some mineral fertilizers and pesticides in agriculture and horticulture, and improper waste storage [105–107]. In the EU, fuel combustion contributed to 72% of Cd and 61% of Pb emissions. The overall emissions of Cd, Pb and Zn in the EU fell significantly between 1995 and 2014, by 59.2% for Cd, 82.5% for Pb and 34.1% for Zn. The reductions were smaller in Poland: 48.1% for Cd, 14.5% for Pb and 23.2% for Zn. Cu emissions in Europe were close to a constant level. Poland is the largest source of Cd (22.2%) and Pb (26.9%) in the EU and the second largest source of Cu (9.1%) and Zn (20.0%) [103].

The aims of the study were as follows:

- to determine the background pollution by SO_2_ and heavy metals in central Poland using bioindication methods
- to evaluate the impact of changes in overall country emissions on environmental pollution expressed as the accumulation of pollutants in plants.

## Study area

**Fig 1:**
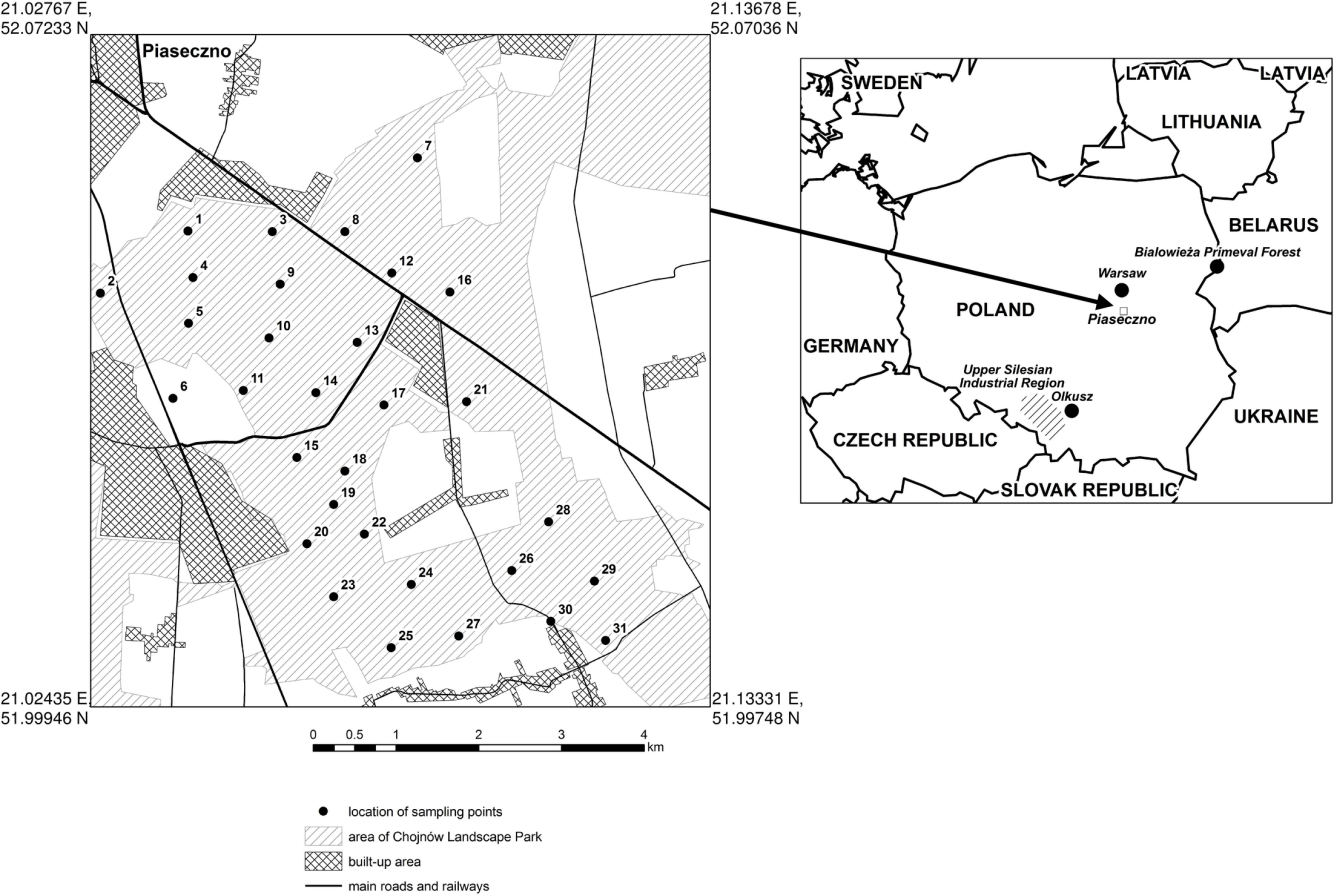
Map of Poland indicating geographical features discussed in the article and the study area.

The Chojnowskie Forests have an area of 10 200 ha and are characterized by a rather high forest density. Mixed fresh coniferous forest with a predominance of pine constitutes the majority of the habitat at 74.1%, with 9.1% oak and 8.6% birch [108]. The forests are located in central Poland beyond the direct impact of large stationary pollution emitters. The air quality of the Chojnowskie Forests is influenced by the city (Warsaw). However, there are no large industrial emitters in the city. The sources of air pollution in the Chojnowskie Forests are large hard coal-fired power stations: 3 combined heat and power stations in Warsaw (total 4266 MW, located approximately 30 km northeast of the Chojnowskie Forests), Kozienice Power Station (2905 MW) 48 km southeast, and the lignite-fired Bełchatów Power Station (5420 MW) situated approximately 148 km southwest of the Chojnowskie Forests. The flue gases of these plants are emitted into the atmosphere through stacks whose heights are 200–300 m. The air pollution levels of the Chojnowskie Forests can be regarded as background air pollution for central Poland [21]. The locations of the study area and sampling points are presented in Fig 1.

The study was carried out on state land which belong to State Forests (Poland). We have obtained the respective agreement from regional office of State Forests for sampling collection in study area (Chojnowskie Forest).

We confirm that the field studies did not involve endangered or protected species.

### Materials and methods

The highest possible standardization of all activities involved in the study was applied; this is necessary to obtain comparable results using bioindication [109]. The Scots pine needles used for the analyses were collected in the second half of March in the years 2006 and 2014 according to the methodology presented by Dmuchowski and Bytnerowicz [21]. In the first year, needles were collected from 15–25-year-old trees, the same trees in both years of the study, from the 2^nd^ to 4^th^ whorl from the top by cutting branches from the outer parts of the canopy. The needles were collected from eight trees growing side by side. From the collected needles, a collection sample was created from each location, taking 40 g from each tree. Only the needles from previous year's growth were used. Total samples from 31 locations were collected. The materials collected were placed in linen bags and dried at 70°C. The dried materials were pulverized in a stainless-steel impact mill (Fritsch 14702) and stored in tightly sealed plastic containers until the time of analysis. Needles were washed for one minute in distilled water before being dried and pulverized. The powdered samples were dry-mineralized in a muffle oven (Naberthern L40/11/P320) using the following time/temperature procedure: 120°C/2 h, 200°C/1 h, 300°C/1 h, and 450°/5 h. The ashes were digested in 30% HCl (Merck Suprapur) and filtered through filter paper [110].

The analyses were performed via flame AAS (Perkin Elmer 1100A) corrected to deuterium background correction using hollow cathode lamps (HCl) and acetylene burners [111]. Three identical subsamples of each sample were processed. Three blanks were run with each batch of samples; thus, each sample was blank-corrected. Total sulfur content was determined using a LECO 132 apparatus [112].

To provide quality control (QC), the elemental content in the plant samples was determined using certified reference materials (Table 4). The obtained results were in close agreement with the certified values. The recovery range was from 94.7% to 106.0%.

**Table 4:** Comparisons of measured and certified concentrations of elements in certified materials.

Element	Certified	Measured	Recovery (%)
Pine needles* –mg kg^-1^ (NIST– 1575a)			
Pb	0.167 ± 0.015	0.173 ± 0.018	103.6
Cd	0.233 ± 0.004	0.222 ± 0.008	- 95.3
Zn	38 ± 2	40 ± 2	105.3
Cu	2.8 ± 0.2	2.9 ± 0.3	103.6
Beech leaves** (BCR 100)			
S (%)	0.269 ± 0.04	0.259 ± 0.05	- 96.3
Cr (mg kg^-1^)	8.0 ± 0.6	7.6 ± 0.5	- 95.0

*National Institute of Standards and Technology (USA)

**European Commission–Institute for Reference Materials and Measurements

### Results and discussion

**Fig 2:**
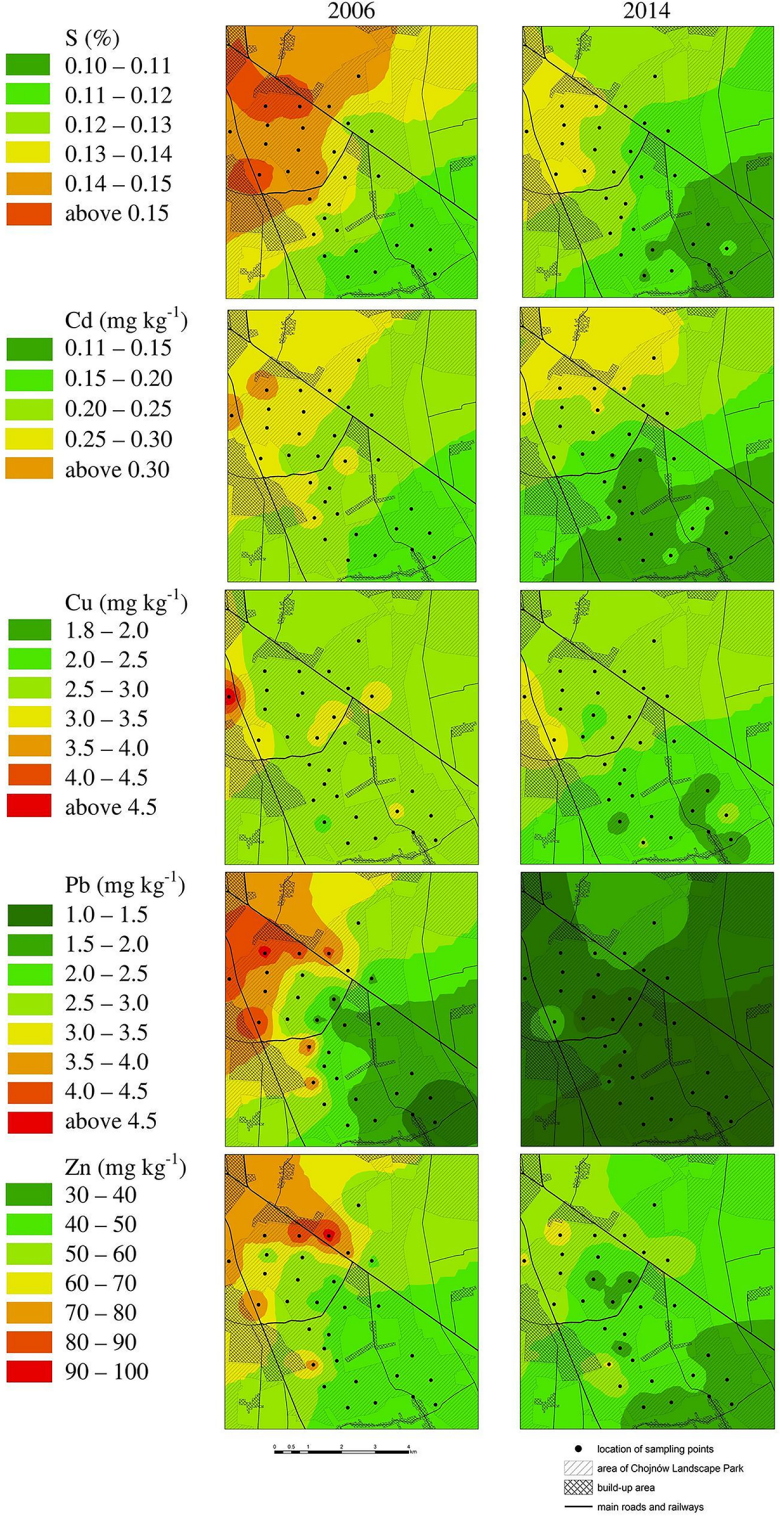
Contamination of the environment with S and metals based on the accumulated concentration of this element in the Scots pine needles in the years 2006 and 2014.

All results for element content and comparisons with other studies concern Scots pine needles from the previous year. The results for metal and SO_2_ content in needles are presented in Table 5 and on the maps with isolines (Fig 2). The distribution and values of isolines suggest a significant decrease in pollution in 2006–2014 for all studied elements. The higher level of pollution in the northern region of the Chojnowskie Forests indicates the influence of the emissions in the Warsaw area. For almost all elements presented in Table 5, the mean content was significantly lower in 2014 than in 2006. Only for the Cu levels was the difference not significant (P > 0.05). The highest relative variability of the content of elements was observed for Pb (high standard deviation in relation to the mean). The lowest variability was observed for S and Cu. The strongest relationship between pairs of elements (Table 6) was observed for Pb and Zn in 2006, which means that these elements are pollutants that almost always spread together in the air. Very strong relationships were also observed between Cd and S, Pb, and Zn in 2006. In 2014, the relationships were quite strong, but weaker in comparison with the year 2006. The strongest relationship between the content of an element in 2006 and the content of an element in 2014 was observed for sulfur (Table 7). This means that a similar spatial pattern of pollution was observed for the sulfur content. The weakest relationship was observed for the Cu content, i.e. that the spatial distribution of the Cu content was different in 2006 and in 2014.

**Table 5:** Sulfur content (%) and metals (mg kg^-1^) in Scots pine needles–descriptive statistics.

	Mean	Median	Minimum	Maximum	Lower quartile	Upper quartile	Standard deviation
S-2006	0.13	0.14	0.11	0.16	0.12	0.15	0.02
S-2014	0.12	0.12	0.10	0.14	0.11	0.13	0.01
Cd-2006	0.23	0.23	0.15	0.33	0.20	0.27	0.05
Cd-2014	0.18	0.18	0.08	0.30	0.13	0.22	0.06
Cu-2006	2.82	2.70	2.20	4.90	2.50	2.90	0.52
Cu-2014	2.53	2.61	1.70	3.78	2.07	2.86	0.52
Pb-2006	2.66	2.20	1.30	4.70	1.80	3.90	1.15
Pb-2014	1.03	0.89	0.29	2.24	0.63	1.40	0.51
Zn-2006	55.87	48.00	40.00	97.00	45.00	70.00	16.37
Zn-2014	45.67	46.92	24.71	66.25	35.61	55.79	11.17

**Table 6:** Spearman’s rank correlation coefficients between the contents of elements in Scots pine needles.

	S	Cd	Cu	Pb
Year 2006				
Cd	0.818*			
Cu	0.164	0.081		
Pb	**0.771**	**0.813**	0.030	
Zn	**0.770**	**0.833**	0.039	**0.903**
Year 2014				
Cd	**0.612**			
Cu	**0.583**	**0.629**		
Pb	**0.600**	**0.711**	**0.714**	
Zn	**0.516**	**0.476**	**0.444**	**0.635**

* Correlation coefficients in bold indicate significant relationships at P < 0.05

**Table 7:** Spearman’s rank correlation coefficients between the contents of the same elements in Scots pine needles in two years (2006 vs. 2014).

S	0.878*
Cd	**0.619**
Cu	0.233
Pb	**0.753**
Zn	**0.689**

* Correlation coefficients in bold indicate significant relationships at P < 0.05

The content of S and metals in needles is affected by air pollution associated with the transport of pollutants from distant and local emission sources, and soil pollution from current and historical deposition. Important is also the different availability of the tested elements from the soil and air.

In 2006, the sulfur content in the needles ranged from 0.112% to 0.159%, with an average of 0.133%. In 2014, the content was approximately 9.9% lower (average 0.120%, range 0.101%–0.137%). The positive correlation between the sulfur content in needles and the concentration of SO_2_ in the air has been known for many years [18,21,24,113]. The level of S is determined by its level in the air and not in the soil. The study area can be recognized as a background area for central Poland [21]. At the beginning of the 1980s in the Białowieża Primeval Forest, the least polluted region in northeastern Poland, the sulfur content in needles ranged from 0.088% to 0.091%, compared to 0.091% to 0.120% in central Poland, while at the same time it averaged 0.071% in Lapland in northern Finland [13,21]. In 2002, the background level amounted to 0.087% in northeastern Poland and 0.109% in central Poland [51]. In a background pollution region in Lapland, the sulfur content in needles was measured to be 0.066% in 2001 and 0.063% in 2007 [67]. Significantly lower levels, ranging from 0.030% to 0.033%, were observed in the Baikal region in Siberia in 2010 [58]. The sulfur content in needles is significantly higher in industrial regions with high SO_2_ emissions, e.g. 0.320% in Finland in 1976–1978 [10] and 0.201% in the Upper Silesian Industrial Region in Poland in 1982 [13], as well as 0.212% in 1991 [25].

**Fig 3:**
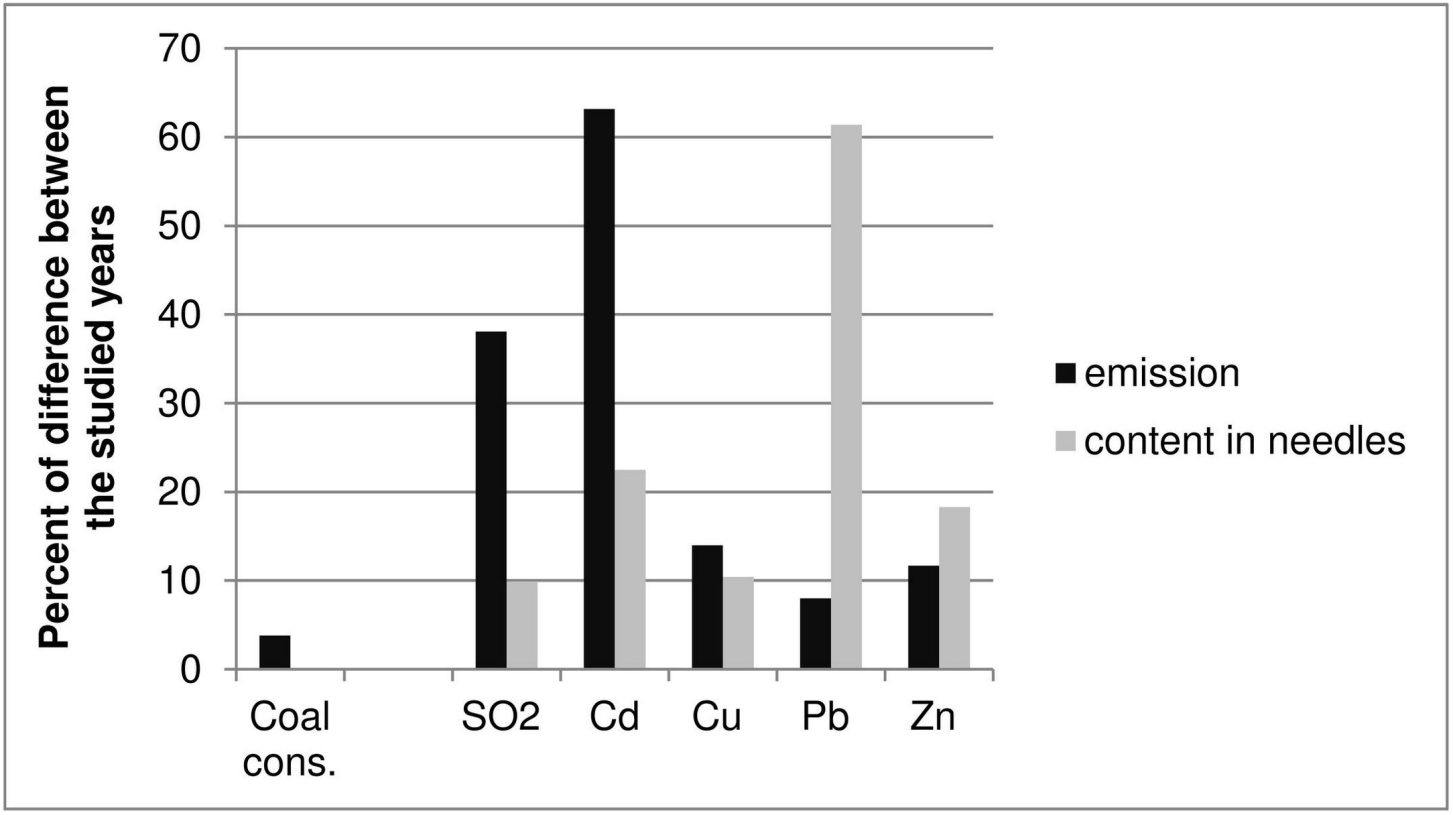
Changes in coal consumption for energy purposes, SO_2_ and metals emissions in Poland and contents of elements in Scots pine needles in the years 2006–2014.

The total SO_2_ emissions in Poland decreased by 38.1% in the years 2006–2014 [103], along with a fall in the needle sulfur content by 9.9% (Fig 3). The reduction of sulfur emissions did not correspond with a change in coal consumption, the combustion of which is the main source of SO_2_ pollution in the air. Rather, it was a result of the installation of gas desulfurization equipment in some power stations. In 2006, only 61% of SO_2_ was retained and neutralized in these devices, while in 2014 this figure was 81.0% [104–105]. The smaller drop in the sulfur content in Scots pine needles compared to the reduction of emissions could have been caused by low local emissions from stoves without filtration devices.

The average Cd content in pine needles in the research area was 0.23 mg kg^-1^ (0.15–0.33 mg kg^-1^) in 2006, and this decreased to 0.18 mg kg^-1^ (0.08–0.30 mg kg^-1^) in 2014 (a 22.5% reduction). In 1982, needles in the Lapland area contained 0.17 mg kg^-1^ Cd, while in the Białowieża Primeval Forest, the least polluted region of Poland, it was 0.22 mg kg^-1^ in 1990 and 0.12 mg kg^-1^ in 2005 [13,59]. In the years 1984–1988 in Norway in a background pollution region, pine needles contained 0.27 mg kg^-1^ Cd [72]. The level of Cd was 0.63 mg kg^-1^ in Slovakia in 1979 [9]. In 2005, in a high-pollution area in Poland near a Pb-Zn smelter, the Cd content in needles reached 5.78 mg kg^-1^ [59] and 2.4 mg kg^-1^ in 2014 [82].

In the period 2006–2014, Cd emissions in Poland decreased by 63.2% in spite of a reduction of coal combustion of 3.8% (coal is the main Cd emission source) [103]. The Cd content in needles fell by 22.5% and thus this did not have a direct connection with changes in Cd emissions or in the coal consumption rate. The content of Cd in needles is affected not only by pollution from current emissions, but also by historical deposition into the soil. Cd emissions and coal consumption in Poland in the years preceding the study were larger. The mean Pb content in needles in the Chojnowskie Forests was 2.66 mg kg^-1^ in 2006 (range 1.30–4.70 mg kg^-1^) and this fell to 1.03 mg kg^-1^ in 2014 (range 0.29–2.24 mg kg^-1^). Earlier in Poland in the area with the least background pollution, pine needles contained significantly higher amounts of Pb: 10.8 mg kg^-1^ in 1982 [13] and 1.29 mg kg^-1^ in 1992 [21]. Background pollution in Slovakia was 5.4 mg kg^-1^ in 1980 [9], and in southern Norway it was 4.0 mg kg^-1^ in 1982, and 1.1 mg kg^-1^ in 1992 [72]. The mean needle content of Pb was significantly higher in highly polluted regions. In the Upper Silesian Industrial Region in Poland, needles contained 54.8 mg kg^-1^ Pb in 1982 [13] and 10.1 mg kg^-1^ in 1992 [21]. In the Warsaw Steelworks region, the mean Pb content was 19.2 mg kg^-1^ in 1991 and, in the proximity of a Pb-Zn smelter in Olkusz, it reached 255 mg kg^-1^ at its peak in 1991 [25]. In the proximity of a Cu-Ni smelter in Lapland, needles had 9.3 mg kg^-1^ of Pb content in 1991 [30], while in Erzurum in Turkey, it was 29.0 mg kg^-1^ on average.

The Pb content in needles in the Chojnowskie Forests decreased by 63.2% on average in the period 2006–2014. The reduction of total Pb emissions in Poland in this period was only 8.0% [103]. The Pb content in the needles is influenced by the Pb content in the soil and the direct inflow from the air. The availability of Pb from the soil increases with the lowering of pH. Soils in coniferous forests in Poland are naturally acidic. Hovmand et al.'s [114] research showed little root uptake of Pb compared to Pb absorbed by the needles from atmospheric deposition. This tendency has also been confirmed in research on other plants [115–118]. Such a high decrease in pollution at the studied sites could be a result of the elimination of leaded gasoline in 2000 [21], which was a decisive factor in local pollution levels. The relatively low decrease in overall Pb emissions was caused by changes in coal combustion, which was reduced by only 3.8% [104–105].

Zinc, unlike the abovementioned metals, is a common element in human and natural environments and plays multiple roles in living organisms. In the study area, the mean Zn content in needles was 55.9 mg kg^-1^ in 2006 (range 40.0–97.0 mg kg^-1^) and this dropped to 45.7 mg kg^-1^ in 2014 (range 24.7–66.2 mg kg^-1^), i.e. by 18.3%. The background Zn level in needles in northeastern Poland was 51 mg kg^-1^ in 1992 [21] and this fell to 34.0 mg kg^-1^ in 2005 [64]. Similar Zn levels were observed in needles in Lapland in 1982 (45 mg kg^-1^) [13]. In 1982, in southern Norway the background content was higher than in Poland and amounted to 75 mg kg^-1^ on average (range 56–99 mg kg^-1^) [72]. The needle Zn content was significantly higher in polluted environments. In the Upper Silesian Industrial Region in Poland, needles contained 164 mg kg^-1^ Zn in 1982 and 174 mg kg^-1^ in 1992 [13,21]. The highest Zn needle contents were found near a Pb-Zn smelter in Olkusz, reaching 548 mg kg-1 [64]. A very high Zn content was also observed in the proximity of an Ni smelter in Lapland in 2000, ranging from 319–1010 mg kg-1 Zn [33]. In the period 2006–2014, global Zn emissions in Poland decreased by 11.7%, and there was a decline in Zn needle content of 18.3%.

Investigation into the Cu content in Scots pine needles is not recognized as a good bioindication method for measuring environmental pollution, because an excessive accumulation of Cu in roots and transport of Cu to upper parts of trees may occur when the demand for this element emerges [21,119]. The Cu content in needles in the Chojnowskie Forests ranged from 2.53 to 2.82 mg kg-1 and this was at the same level as the background content in the preceding years in the Białowieża Primeval Forest in Poland, as well as in Slovakia, in southwestern Finland and in Lapland [9,13,22]. The needle Cu content was at a level that is typical for plants [120].

## Conclusion

Environmental pollution by sulfur and metals in the Chojnowskie Forests, except for the northern part, mean that it can be recognized as a background area for central Poland. In the northern part, there has been a noticeable influence of pollution from the Warsaw area.

The changes in the contents of the analyzed elements in Scots pine needles in the period 2006–2014 (in the previous year’s needles) in the Chojnowskie Forests can be arranged in a decreasing series:

Pb>Cd>Zn>Cu>S

The changes in the total emission of SO_2_ and metals in Poland can be arranged as follows:

$$Cd>SO_{2}>Cu>Zn>Pb$$

The changes in Scots pine needle pollution content from sulfur and heavy metals were not compatible with the levels of changes in global emissions in Poland. The causes of this result could include the availability of the emitted pollution forms for plants, the spread of contamination at short distances from emission sources, historical environmental contamination in the study area, the ability of plants to take up nutrients from soil and the biological fixation in soil of inactive forms.

The changes in emissions of each element should be considered separately. The highest decrease in pollution in needles was found for Pb (61.4%), much greater than the reduction of the global emissions (8.0%). The reason for this could be soil pollution depletion derived from historical emissions from leaded gasoline. The lack of compatibility of emissions and contents in plants does not disprove bioindication methods. The estimation of pollution levels in plants reflects their incorporation in biological processes rather than air or soil pollution levels.

Coal combustion is the main source of emissions of sulfur and other studied elements. In 2006–2014, a 3.8% decrease in coal consumption was observed. This was significantly lower than the reduction in emissions. This is probably the result of the installation of gas desulfurization technologies in power stations and higher levels of reduction of pollutant emissions in neighboring countries and thus the reduction of pollution from cross-border transport.
